# Facile Synthesis of Asymmetric aza-Boron Dipyrromethene Analogues Bearing Quinoxaline Moiety

**DOI:** 10.3390/molecules28247940

**Published:** 2023-12-05

**Authors:** Ru Feng, Zuoxu Chen, Yue Wang, Jianming Pan, Soji Shimizu

**Affiliations:** 1 School of Chemistry and Chemical Engineering, Jiangsu University, Zhenjiang 212013, China; fengru@ujs.edu.cn (R.F.); chenzuoxu1112@163.com (Z.C.); yue.wang@ujs.edu.cn (Y.W.); 2 Department of Applied Chemistry, Graduate School of Engineering, Kyushu University, Fukuoka 819-0395, Japan; 3 Jiangsu Chunlan Clean Energy Academy Co., Ltd., Taizhou 225300, China; 4 Jiangsu Agrochem Laboratory Co., Ltd., Changzhou 213022, China; 5 Center for Molecular Systems (CMS), Kyushu University, Fukuoka 819-0395, Japan

**Keywords:** aza-BODIPY, quinoxaline, hydrogen bonding, fluorescence

## Abstract

An asymmetric aza-BODIPY analogue bearing quinoxaline moiety was synthesized via a titanium tetrachloride-mediated Schiff-base-forming reaction of 6,7-dimethyl-1,4-dihydroquinoxaline-2,3-dione and benzo[*d*]thiazol-2-amine. This novel aza-BODIPY analogue forms a complementary hydrogen-bonded dimer due to the quinoxaline moiety in the crystal structure. It also shows intense absorption and fluorescence, with fluorescence quantum yields close to unity. The electrochemical measurements and the DFT calculations revealed the presence of the low-lying HOMO, which benefits their potential applications as an electron-transporting material.

## 1. Introduction

Boron dipyrromethenes (BODIPYs) and their *meso*-nitrogen-substituted analogues called aza-BODIPYs have received considerable research attention owing to their fascinating optical properties and potential applications, such as bioimaging [1,2,3,4,5,6,7], chemosensors [8,9,10,11], laser dyes [12], sensitizers for photodynamic [13,14,15] and photothermal therapies [16,17], and optoelectronic materials for light-emitting diodes [18,19,20] and organic photovoltaics [10,21]. Despite their excellent chemical and photophysical properties, the fluorescent emission of most BODIPY derivatives is virtually quenched in the solid state due to the small Stokes shifts and strong intermolecular π-π stacking interactions enhanced by the symmetric, planar structure [22,23,24,25,26]. To address these drawbacks, considerable research efforts have been devoted to developing BODIPY analogues with asymmetric structures, for which large Stokes shifts can be expected, owing to the structural changes between the ground and excited states. In addition, asymmetric BODIPY analogues tend to take head-to-tail stacking patterns in the solid state to minimize dipolar interactions [5,27,28,29,30,31,32,33,34,35,36,37,38,39]. However, compared with the conventional symmetric BODIPYs, there is still a great challenge for the synthesis of asymmetric BODIPYs.

**Chart 1:**
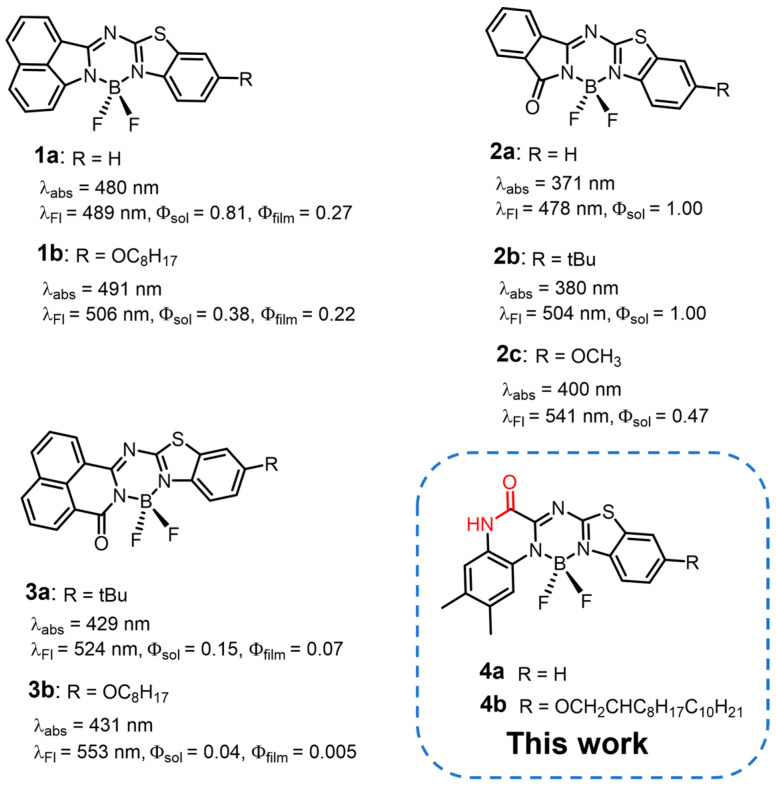
Structures of asymmetric aza-BODIPYs synthesized via a Schiff-base-forming reaction.

Recently, we have reported a Schiff-base-forming reaction of azaarylamines and diketopyrrolopyrrole, in which the lactam moieties of diketopyrrolopyrrole were successfully converted to aza-BODIPY structures in the presence of titanium tetrachloride (TiCl_4_) and triethylamine (NEt_3_) [40,41]. A series of asymmetric aza-BODIPYs were also synthesized from monolactams based on the TiCl_4_-based method. The asymmetric compounds exhibited moderately high fluorescence quantum yields of 0.13–0.27 (Chart 1, **1a**,**b**) [39]. Lu et al. revealed that 1,8-naphthalenimide, isoindole-1,3-dione, and their derivatives can also be converted to asymmetric aza-BODIPYs in simple ethanol reflux with azaarylamines, followed by boron complexation (Chart 1, **3a**,**b**) [29]. The aza-BODIPYs thus synthesized, which have a ketone moiety, showed intense fluorescence in solution and moderate solid-state emission (Φ_F_ = 0.005–0.22) due to the asymmetric structures. Jiao et al. reported the synthesis of similar asymmetric aza-BODIPYs from isoindole-1,3-dione based on the TiCl_4_-mediated method (Chart 1, **2a**–**c**) [28]. In contrast to conventional symmetric BODIPYs, these asymmetric aza-BODIPYs exhibited large Stoke shifts and high photostability. The above-mentioned studies indicate that the Schiff-base-forming reaction is a versatile method of synthesizing asymmetric aza-BODIPYs with unique optical and electronic properties.

To broaden the scope of the TiCl_4_-mediated Schiff-base-forming reaction for the synthesis of asymmetric aza-BODIPYs, herein, we investigated the synthesis of novel asymmetric aza-BODIPYs (**4a** and **4b**) bearing a quinoxaline moiety. The target compounds were synthesized from 6,7-dimethyl-1,4-dihydroquinoxaline-2,3-dione and 2-aminobenzothiazole. These asymmetric aza-BODIPYs showed intense absorption and fluorescence in solution. In the crystal structure of **4a**, a complementary hydrogen-bonded dimer and the J-type arrangement of the dimer were observed in the packing diagram.

## 2. Results and Discussion

### 2.1. Synthesis and Characterization

**Scheme 1:**
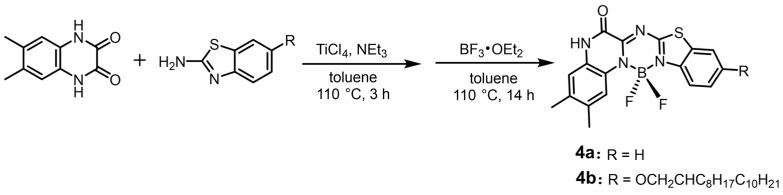
Synthesis of **4a** and **4b**.

The Schiff-base-forming reaction of 6,7-dimethyl-1,4-dihydroquinoxaline-2,3-dione and benzo[*d*]thiazol-2-amine in the presence of TiCl_4_ and NEt_3_ and subsequent boron complexation provided the target asymmetric aza-BODIPYs, **4a** and **4b** (Scheme 1). The aza-BODIPY **4a** was obtained in a higher yield of 42% than **4b** (27%) despite its lower solubility than **4b**. We characterized **4a** and **4b** by NMR spectroscopy (Figures S1–S4).

**Figure 1:**
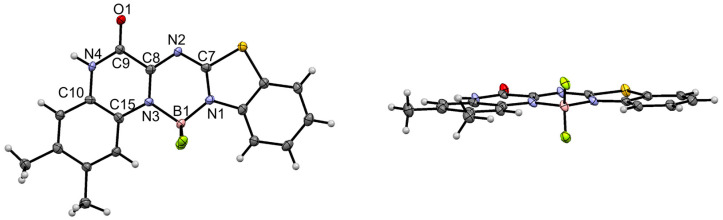
X-ray crystal structure of **4a** (top view: left; side view: right). The thermal ellipsoids were scaled at 50% probability.

**Figure 2:**
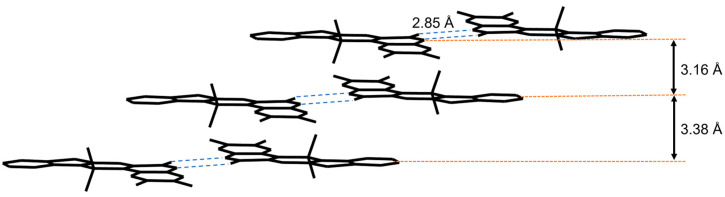
Molecular packing diagram of **4a**. Hydrogen bonds are shown as blue dashed lines. The interplanar distances between the mean planes of molecules excluding the two fluorine atoms are shown.

The structure of **4a** was unambiguously elucidated by X-ray crystallography. In a unit cell, an acetonitrile molecule was also found as a solvent molecule. The single crystal of **4a** was obtained via the slow diffusion of acetonitrile into a chloroform solution of **4a** (Figure 1). The aza-BODIPY **4a** features a highly coplanar structure in the crystal structure. The C9–O1 bond length of **4a** (1.228(3) Å) is a typical C=O bond length (ca. 1.22 Å). The longer bond distance of B1–N3 (1.577(4) Å) than that of B1–N1 (1.543(4) Å) implies the asymmetric structure of **4a**. Similar trends have been reported for the B-N bonds in other asymmetric amido/imino BF_2_ complexes [28,29,31,42,43]. In the unit cell, two molecules were stacked with each other to form π-π stackings (Figure S5). The amide unit of the quinoxaline moiety formed a hydrogen bond with the neighboring molecule with N–H⋯O distance of 2.85 Å (Figure 2). The hydrogen bonds mean that the molecules are nearly parallel to each other. In the molecular packing diagram, hydrogen-bonded dimers are slip-stacked in a J-type arrangement. The interplanar distances between the mean planes of the next neighbors are 3.16 and 3.38 Å (Figure 2). The above results indicate that hydrogen bonding is critical for influencing molecular packing and determining the crystal structure.

**Figure 3:**
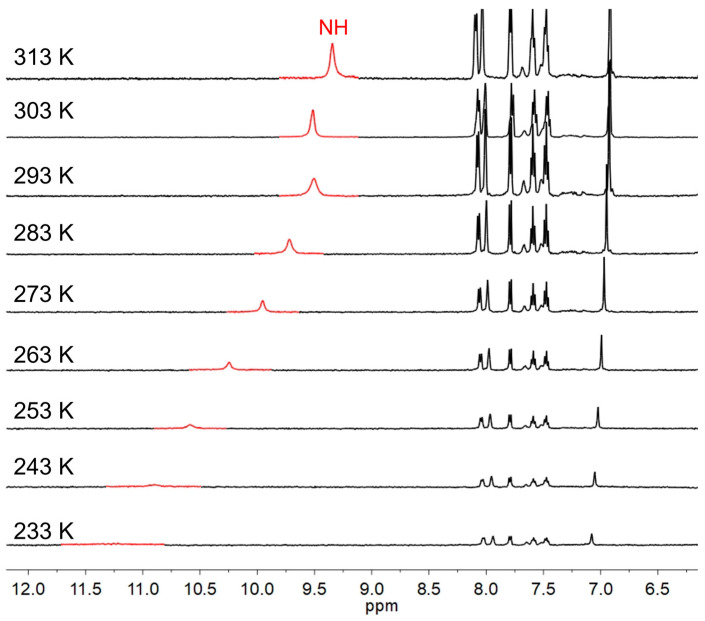
Variable-temperature ^1^H NMR spectra of **4a** in 1,1,2,2-tetrachloroethane-*d*_2_. The NH proton signals are highlighted in red.

To give a deep insight into the hydrogen-bonding interactions in solution, temperature-dependent chemical shifts of the ^1^H NMR spectra of **4a** were investigated in 1,1,2,2-tetrachloroethane-*d*_2_. As shown in Figure 3, the downfield shift of the NH proton signal from 9.39 to 11.23 ppm, together with significant broadening, was observed when the temperature decreased from 40 °C to –40 °C due to the formation of intermolecular hydrogen bonds at low temperatures.

### 2.2. Photophysical Properties

**Figure 4:**
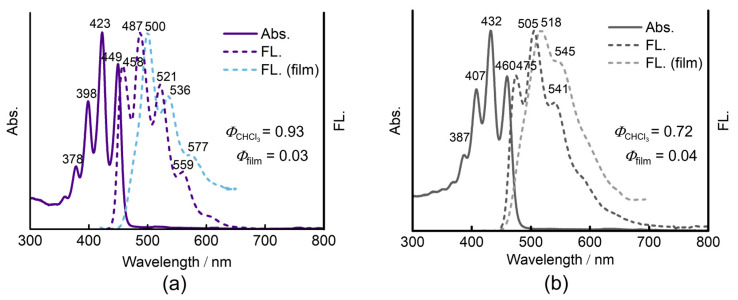
UV/vis absorption (solid line) and fluorescence (dashed line) spectra of (**a**) **4a** and (**b**) **4b** in CHCl_3_.

**Chart 2:**
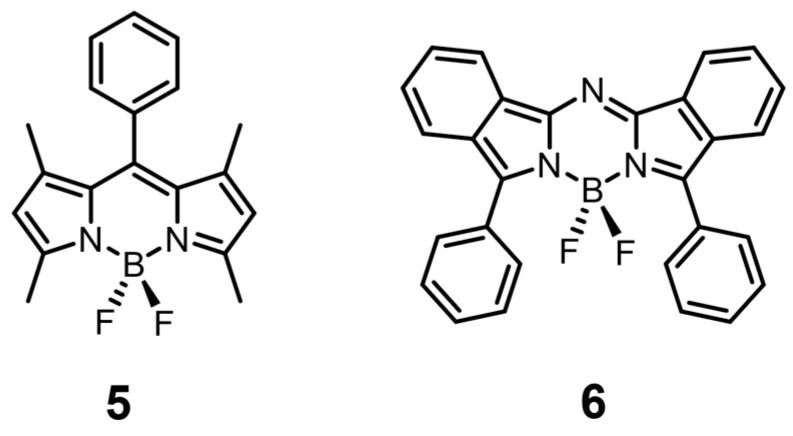
Structures of BODIPY **5** and aza-BODIPY **6**.

The photophysical properties of **4a** and **4b** were measured in chloroform solution and film (Figure 4). The details are summarized in Table 1. The aza-BODIPY **4a** exhibits absorption with distinctive vibronic structures comprising the less intense 0-0 band at 449 nm and the 0-1 vibronic band at 423 nm as a main band in the visible region. A similar absorption spectral profile with the intense 0-1 vibronic band at 460 nm is also observed for **4b**. The molar absorption coefficients are 1.24 × 10^4^ M^−1^cm^−1^ and 1.97 × 10^4^ M^−1^cm^−1^ for **4a** and **4b** in tetrahydrofuran solution, respectively (Figure S8). Despite the aza-BODIPY-like structure, the absorption spectra of **4a** and **4b** exhibit blueshifts from those of the conventional BODIPY **5** with maximum wavelength (λ_max_) at 500 nm and aza-BODIPY **6** with λ_max_ at 713 nm [44,45] (Chart 2). As detailed in the following section, the DFT calculations revealed that the blueshifts can be ascribed to the different frontier molecular orbital (MO) distribution patterns from those of regular BODIPYs. 

The aza-BODIPYs **4a** and **4b** display intense fluorescence at 458 and 475 nm with small Stokes shifts of 438 and 687 cm^−1^, respectively, as a mirror image to the absorption spectra. The Φ_F_ values are almost unity for **4a** (0.93) and 0.72 for **4b**. The slightly smaller Φ_F_ value of **4b** is mainly due to the structural flexibility arising from the long alkoxy chain, which enhances the nonradiative decay processes.

In contrast to the reported asymmetric aza-BODIPYs such as **1a** and **1b**, which exhibit moderate quantum yields in the film state (0.27 for **1a** and 0.22 for **1b**), the fluorescence of **4a** and **4b** is virtually quenched in the film state, exhibiting Φ_F_ values as law as ca. 0.03–0.04. In the film state, the 0-0 emission band disappeared due to self-absorption. The significant overlap of the absorption and emission due to the small Stokes shifts causes the self-absorption quenching in the solid state.

The impact of pH values on the stability of **4a** was also examined (Figure S9). The absorption and fluorescence spectral profiles of **4a** in acetonitrile do not change in the range from pH = 2 to pH = 11. At a higher pH than pH = 12, both absorption and fluorescence spectra showed redshifts, and the fluorescence spectra became broad and structureless. These spectral changes are probably due to the deprotonation of the amide unit of the quinoxaline moiety. The observed high stability of **4a** over the wide range of pH benefits its use under physiological conditions.

### 2.3. Electrochemical Properties

**Figure 5:**
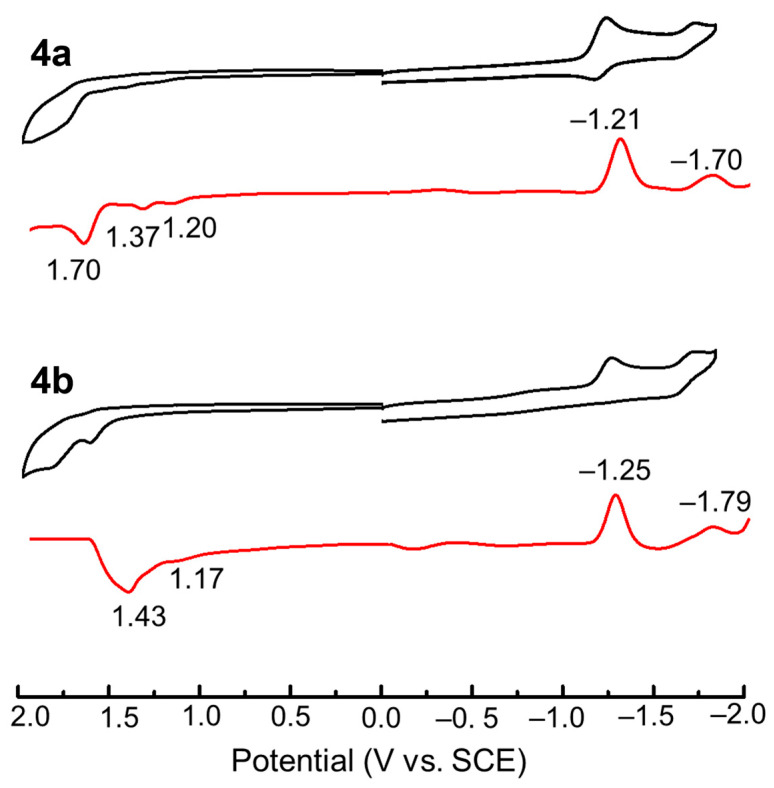
CV (black) and DPV (red) of **4a** (top) and **4b** (bottom) (0.5 mM) in CH_2_Cl_2_ containing 0.1 M TBAP as a supporting electrolyte (scan rate: 100 mV^−1^ for CV; pulse amplitude: 0.05 V, pulse width: 0.2 s for DPV).

Cyclic voltammetry (CV) and differential pulse voltammetry (DPV) measurements were performed in dichloromethane containing 0.1 M tetrabutylammonium perchlorate (TBAP) as a supporting electrolyte (Figure 5). Table 1 summarizes the redox potentials and experimental LUMO and HOMO energy levels estimated from the onset of the first reduction waves and the optical band gaps and LUMO values, respectively. The estimated LUMO (−3.66 eV for **4a** and −3.63 eV for **4b**) and HOMO (−6.29 eV for **4a** and −6.16 eV for **4b**) values are significantly lower than those of the widely used electron-transporting material, Alq_3_ (aluminum tris(8-hydroxyquinolinnate)) (−3.0 eV for LUMO and −5.7 eV for HOMO), implying their potential applications for such a purpose in organic light-emitting diodes.

### 2.4. Theoretical Calculations

**Figure 6:**
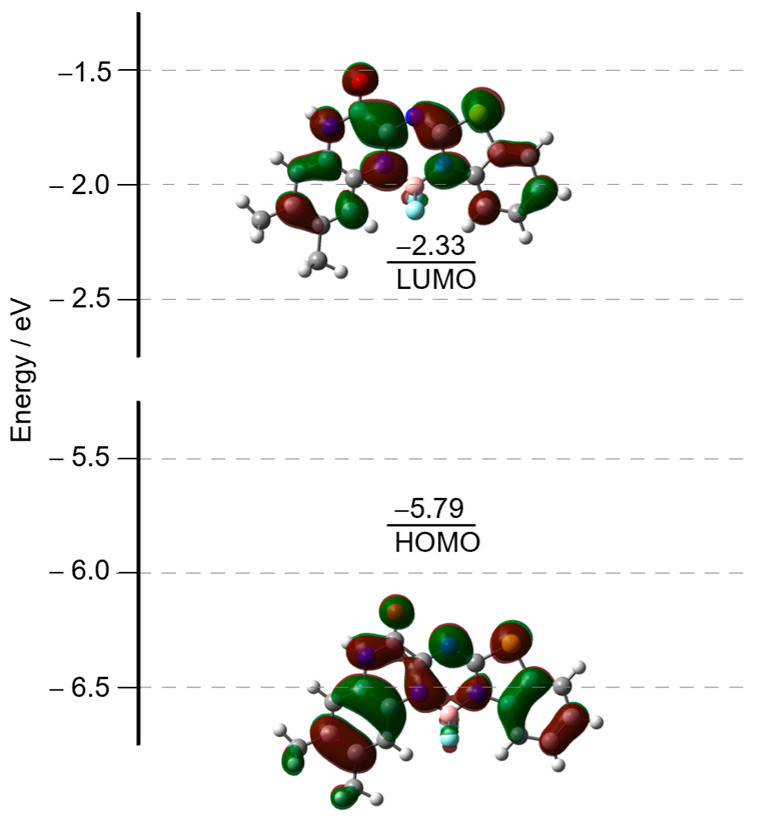
Frontier molecular orbitals of **4** (B3LYP/6-31G(d)).

**Figure 7:**
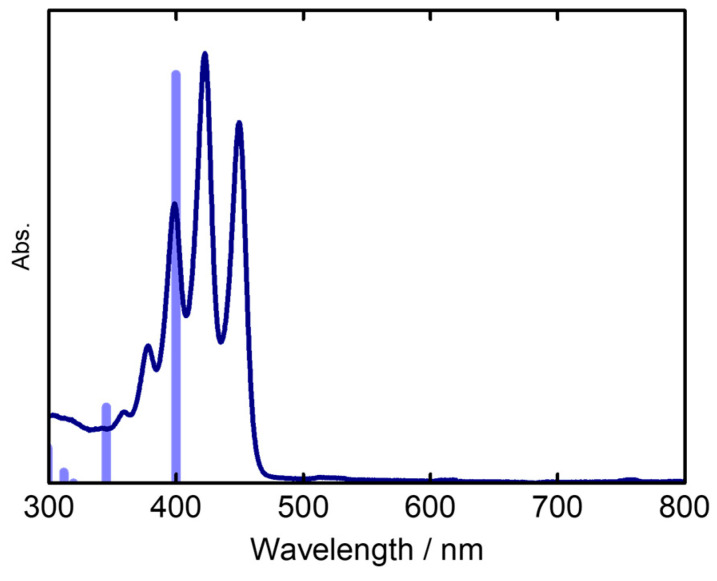
Theoretical absorption bands (blue bars) at the B3LYP/6-31G(d) level and the observed absorption spectrum of **4a** in CHCl_3_ (blue line).

Density functional theory (DFT) and time-dependent (TD)DFT calculations on a model structure of **4** at the B3LYP/6-31G(d) level were conducted to give a detailed insight into the electronic structures (Figure 6 and Figure 7). The TDDFT calculations assign the main absorption band as a HOMO–LUMO transition (Table 2). The TDDFT calculations revealed the wavelength of the S1 excited state at 400 nm with the oscillator strength of 0.46. The S1 state mainly comprises the HOMO-to-LUMO transition. Although the transition energy is slightly overestimated, the TDDFT calculation reproduced the observed absorption spectrum. Because of the absence of an *s*-indacene-like conjugated system in the structure of **4**, the HOMO and LUMO distribution patterns of **4** are different from those of regular aza-BODIPYs, exhibiting larger MO coefficients of the HOMO on the meso-nitrogen atom compared with the LUMO. Therefore, the HOMO is more stabilized than the LUMO by the electron-deficient nitrogen atom at the meso-position. The observed blueshifts of the absorption spectrum of **4** can be explained by the resultant wide HOMO–LUMO gap.

## 3. Materials and Methods

### 3.1. Instrumentation and Measurements

Electronic absorption spectra were recorded on a JASCO V-770 spectrophotometer. Fluorescence spectra were recorded on an SPEX Fluorolog-3-NIR spectrometer (HORIBA) with an NIR-PMT R5509 photomultiplier tube (Hamamatsu). Absolute fluorescence quantum yields were measured using a Hamamatsu Photonics C9920-03G calibrated integrating sphere system with self-absorption correction. The thin films for solid fluorescent measurement were fabricated via spin-coating a solution of **4a** and **4b** in CHCl_3_, respectively. ^1^H NMR spectra were recorded on a JEOL JNM-ECX500 spectrometer (operating at 495 MHz for ^1^H) using a residual solvent as an internal reference for ^1^H (δ = 7.26 ppm for CDCl_3_). CV and DPV measurements were conducted in a CH_2_Cl_2_ solution containing 0.1 M TBAP and 0.1 M samples with a scan rate of 0.1 V s^−1^ under a nitrogen atmosphere. A glassy carbon electrode and a platinum wire were used as the working and counter electrodes, respectively. A saturated calomel electrode (SCE) was used as a reference electrode. The voltammogram display follows the IUPAC convention. Preparative separations were performed using silica gel column chromatography (KANTO Silica Gel 60 N, spherical, neutral, 40–50 mm). All reagents and solvents used for reactions were of commercial reagent grade and were used without further purification unless noted otherwise. All solvents used in optical measurements were of commercial spectroscopic grade.

### 3.2. Crystallographic Data Collection and Structure Refinement

Suitable crystals of **4a** for X-ray analysis were obtained from the vapor diffusion of acetonitrile into a chloroform solution of **4a**. Data collection was carried out at −173 °C on a Rigaku Saturn724 diffractometer with MoKα radiation. The structure was solved via a direct method (SHELXT) and refined using a full-matrix least-squares technique (SHELXL). CCDC 2299504 contains the supplementary crystallographic data for this paper. These data can be obtained free of charge via www.ccdc.cam.ac.uk/data_request/cif (accessed on 1 November 2023), or by emailing data_request@ccdc.cam.ac.uk, or by contacting The Cambridge Crystallographic Data Centre, 12 Union Road, Cambridge CB2 1EZ, UK; fax: +44-1223-336033.

### 3.3. Computational Methods

The Gaussian 16, Revision A.03 [46] software package was used to carry out DFT and TDDFT calculations at the B3LYP/6-31G(d) level of theory. Structural optimizations were performed on model compounds.

### 3.4. Synthesis 

**4a**: 6,7-Dimethyl-1,4-dihydroquinoxaline-2,3-dione (48 mg, 0.25 mmol) and benzo[*d*]thiazole-2-amine (169 mg, 1.13 mmol) were added to dry toluene (6 mL), and the mixture was refluxed at 110 °C. Then, titanium tetrachloride (TiCl_4_) (0.15 mL, 1.38 mmol) and triethylamine (NEt_3_) (0.5 mL, 3.63 mmol) were added to the reaction mixture. After the imine formation was confirmed by TLC analysis in ca. 3 h, borontrifluoride etherate (BF_3_·OEt_2_) (0.48 mL, 3.98 mmol) was added. After refluxing for 14 h, the reaction mixture was added to water and extracted with CHCl_3_. The combined organic extracts were dried over sodium sulfate and concentrated in vacuo to provide yellow solids. The crude compound was chromatographed on a silica gel column using ethyl acetate/hexane = 1:3 to 1:1 (*v*/*v*), then ethyl acetate) to afford **4a** as yellow solids (48 mg, 54%).

^1^H NMR (495 MHz, CDCl_3_, 298 K): δ [ppm] = 10.12 (br, 1H), 8.13 (d, *J* = 9.0 Hz, 1H), 8.07 (s, 1H), 7.78 (d, *J* = 7.5 Hz, 1H), 7.60 (dt, *J*_1_ = 9.0 Hz, *J*_2_ = 1.0 Hz, 1H), 7.47 (dt, *J*_1_ = 9.0 Hz, *J*_2_ = 1.0 Hz, 1H), 7.02 (s, 1H), 2.40 (s, 3H), 2.35 (s, 3H).

**4b**: **4b** was synthesized and purified in a similar manner to **4a**, using 6,7-dimethyl-1,4-dihydroquinoxaline-2,3-dione (48 mg, 0.25 mmol), 6-((2-octyldodecyl)oxy)benzo[*d*]thiazol-2-amine (503 mg, 1.13 mmol), TiCl_4_ (0.15 mL, 1.38 mmol), NEt_3_ (0.5 mL, 3.63 mmol), and BF_3_·OEt_2_ (0.48 mL, 3.98 mmol). The crude mixture was chromatographed on silica gel column (ethyl acetate/hexane = 1:5 (*v*/*v*)) to afford **4b** as yellow solids (27 mg, 16%).

^1^H NMR (495 MHz, CDCl_3_, 298 K): δ [ppm] = 11.33 (br, 1H), 8.05(s, 1H), 8.00 (d, *J* = 9.5 Hz, 1H), 7.22 (d, *J* = 2.0 Hz, 1H), 7.17–7.15 (dd, *J*_1_ = 9.5 Hz, *J*_2_ = 2.0 Hz, 2H), 7.14 (s, 1H), 3.90 (d, *J* = 5.5 Hz, 1H), 2.39 (s, 3H), 2.35 (s, 3H), 1.82 (m, 1H), 1.48–1.28 (m, 32H), 0.89–0.86 (m, 6H).

## 4. Conclusions

In summary, we successfully converted 6,7-dimethyl-1,4-dihydroquinoxaline-2,3-dione as a bislactam precursor to the asymmetric aza-BODIPYs by the Schiff-base-forming reaction. The novel aza-BODIPYs exhibit intense fluorescence in solution, whereas the fluorescence emission is nearly quenched in the film state due to the self-absorption. The crystal structure of **4a** and its molecular packing diagram reveal that **4a** forms a complementary hydrogen-bonded dimer with the next neighbor, and the dimer is further assembled into J-aggregates. The low-lying HOMO based on the electrochemical measurements indicates the potential applications of **4** as an electron-transporting material. An investigation along these lines is being intensively carried out in our laboratory.

## Figures and Tables

**Table 1:** Summary of optical and electrochemical properties of **4a** and **4b**.

							Solution	Film
Compd.	*E*_ox_^a^ [V]	*E*_red_^a^ [V]	*E*_LUMO_^b^ [eV]	*E*_g_^opt c^ [eV]	*E*_HOMO_^d^ [eV]	*λ*_abs_ [nm]	*λ*_em_ [nm]	*λ*_em_ [nm]
**4a**	1.20	–1.21	–3.66	2.63	–6.29	423	487	500
**4b**	1.17	–1.25	–3.63	2.53	–6.16	432	505	518

^a^ Determined by DPV (0.1 M TBAP in CH_2_Cl_2_ as a supporting electrolyte at a scan rate of 100 mV s^−1^). ^b^ *E*_LUMO_ = −($E_{onset}^{red}$ + 4.8) [eV]. ^c^ Estimated from absorption spectra. ^d^ *E*_HOMO_ = *E*_LUMO_ − *E*_g_ [eV].

**Table 2:** Summary of the TDDFT calculation of **4a** at the B3LYP/6-31G(d) level.

Wavelength (nm)	Osc. Strength	Major Contribs
400	0.46	HOMO → LUMO (99%)
344	0.10	H–3 → LUMO (96%)

## Data Availability

The data that support the findings of this study are available from the corresponding author upon request.

## Supplementary Materials

The following supporting information can be downloaded at: https://www.mdpi.com/article/10.3390/molecules28247940/s1, Figures S1–S4. ^1^H NMR and ^1^H-^1^H COSY spectra of **4a** and **4b** in CDCl_3_. Figure S5: (a) Unit cell of the X-ray crystal structure and (b) hydrogen bonding dimer of **4a** in the crystal structure. Hydrogen bonds are shown as dashed lines. Table S1: Crystallographic data of **4a**. Figure S6: X-ray crystal structure of **4a** with labels. Table S2: Bond length for **4a**. Table S3: Bond angels for **4a**. Figure S7: UV/vis absorption spectra of **4a** in various solvents. Figure S8: UV/vis absorption spectra of **4a** (a) and **4b** (b) in THF at different concentrations. Figure S9: UV/vis absorption and fluorescence spectra of **4a** at various pH values in CH_3_CN solution.
