# Supplementary material for: Facile Synthesis of Asymmetric aza-Boron Dipyrromethene Analogues Bearing Quinoxaline Moiety
Source: Molecules. 2023 Dec 5;28(24):7940. doi: 10.3390/molecules28247940 (PMC10745853; doi:10.3390/molecules28247940)
Supplement: Supplementary file 1 [file molecules-28-07940-s001.zip › molecules-2689382-supplementary.pdf]

Supporting information

Facile synthesis of asymmetric aza-BODIPY analogues bearing quinoxaline moiety

Ru Feng [1,2,3,*], Zuoxu Chen [1], Yue Wang [1,4], Jianming Pan [1,*] and Soji Shimizu [2,5,*]

[1] School of Chemistry and Chemical Engineering, Jiangsu University, Zhenjiang 212013, China; fengru@ujs.edu.cn (R.F.); chenzuoxu1112@163.com; (Z.C.); yue.wang@ujs.edu.cn (Y.W.)
[2] Department of Applied Chemistry, Graduate School of Engineering, Kyushu University, Fukuoka 819-0395, Japan
[3] Jiangsu Chunlan Clean Energy Academy Co., Ltd., Taizhou 225300, Jiangsu, China
[4] Jiangsu Agrochem Laboratory Co., Ltd., Changzhou 213022, Jiangsu, China
[5] Center for Molecular Systems (CMS), Kyushu University, Fukuoka 819-0395, Japan
* Correspondence: pjm@ujs.edu.cn (J.P.); ssoji@cstf.kyushu-u.ac.jp (S.S.)

Table of Contents

i. NMR spectra
ii. Crystallographic data
iii. Photophysical properties

i. NMR spectra

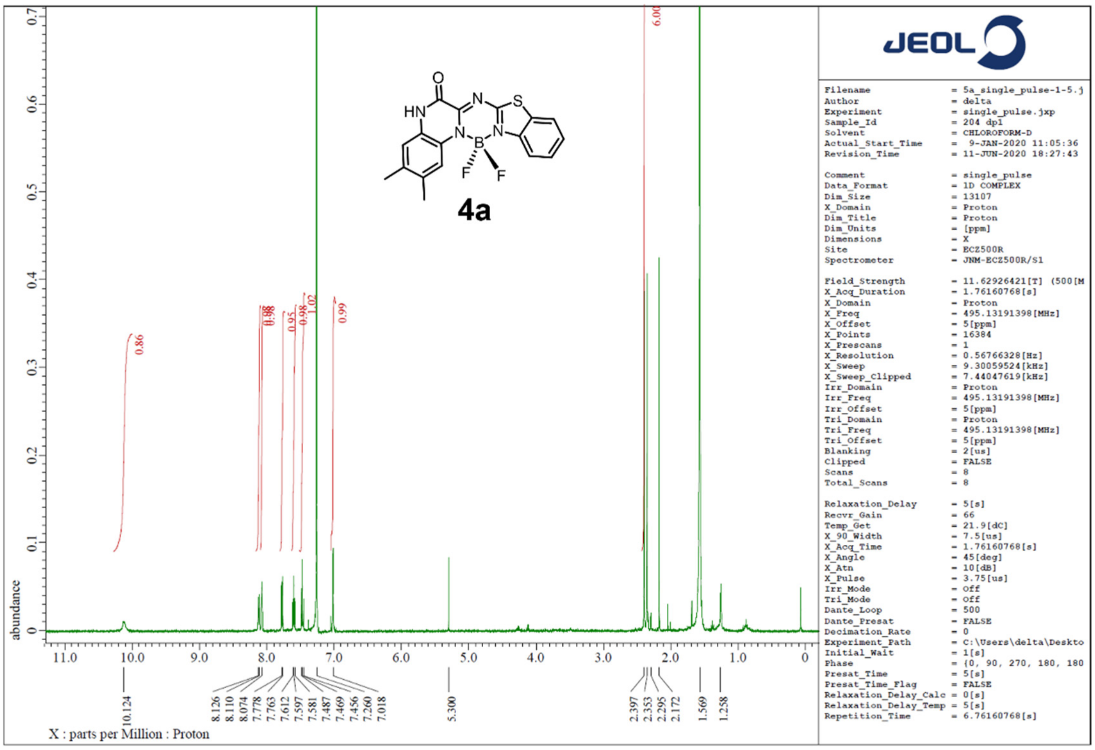

Figure S1. ^{1}H NMR spectrum of **4a** in $CDCl_3$.

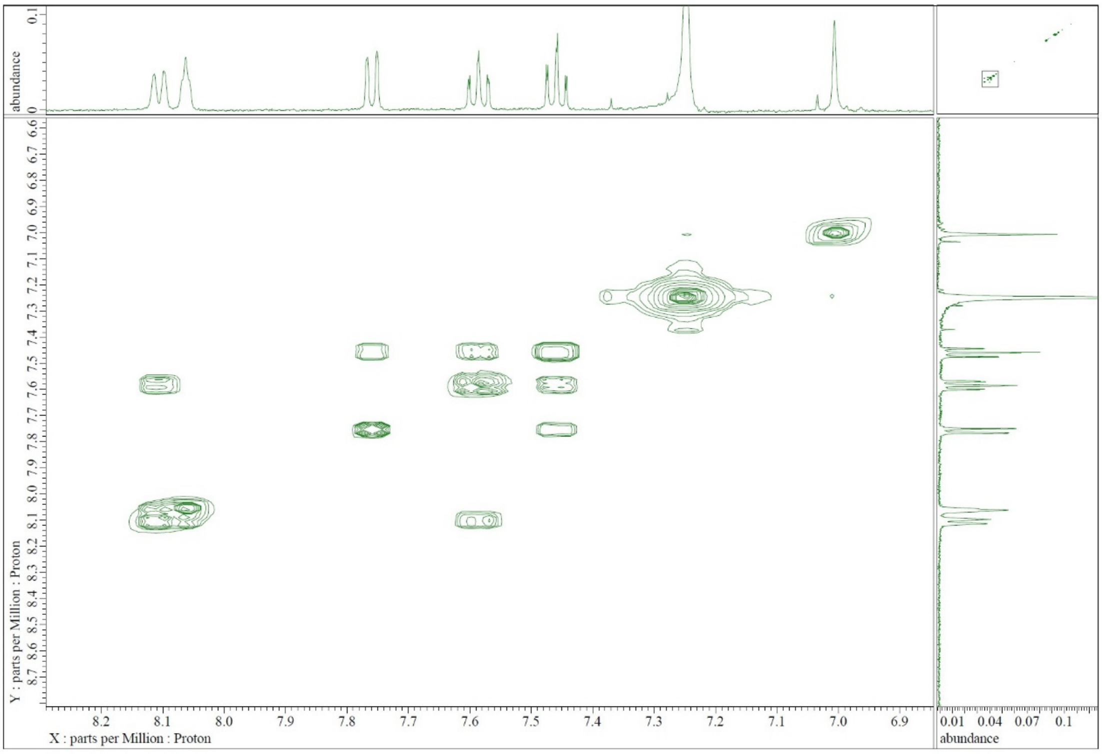

Figure S2. ^{1}H-^{1}H COSY spectrum of **4a** in $CDCl_3$.

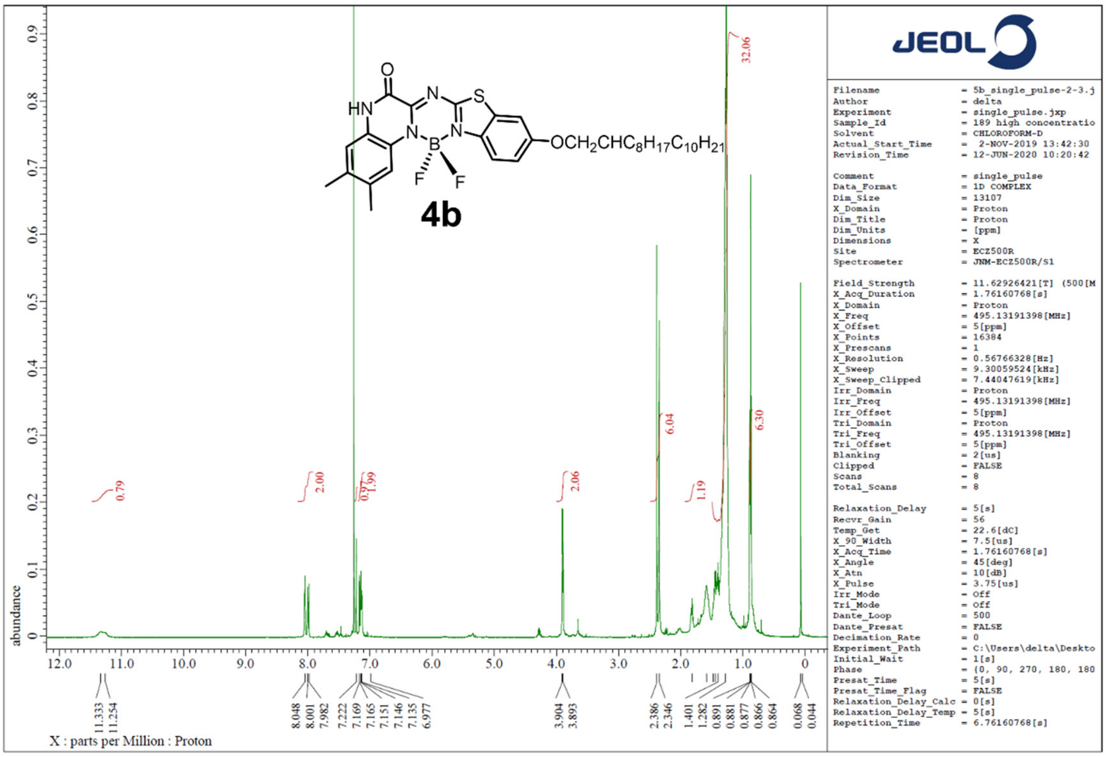

Figure S3. ^{1}H NMR spectrum of **4b** in $CDCl_3$.

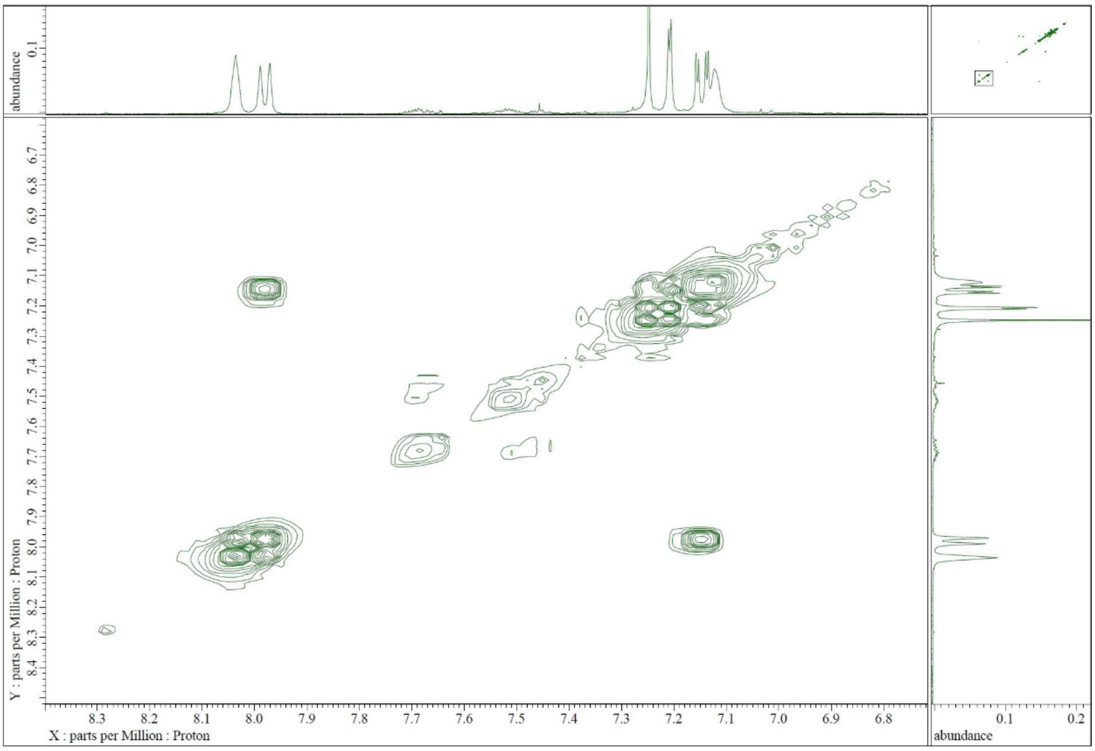

Figure S4. ^{1}H-^{1}H COSY spectrum of **4b** in $CDCl_3$.

ii. Crystallographic data

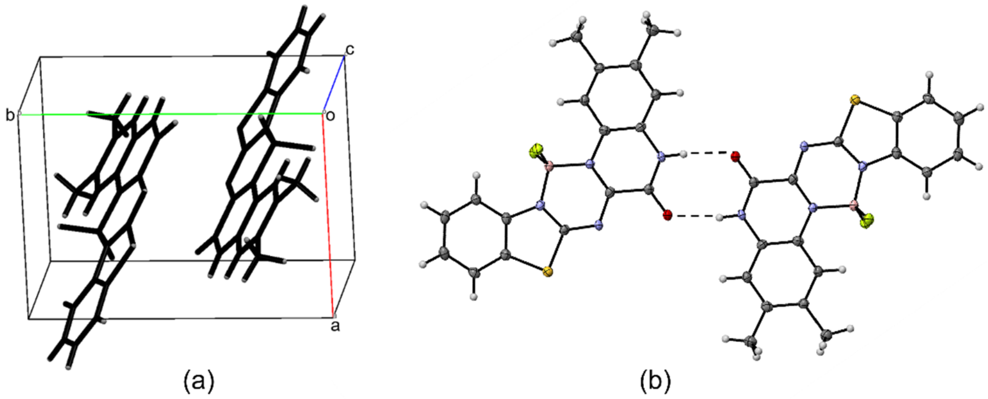

Figure S5. (a) Unit cell of the X-ray crystal structure and (b) hydrogen-bonded dimer of **4a** in the crystal structure. Hydrogen bonds are shown as dashed lines.

Table S1. Crystallographic data of **4a**.

Chemical formula	$C_{17}H_{13}BF_2N_4OS$, C_2H_3N
*M*r	411.24
Crystal system, space group	Triclinic, *P*-1 (no. 2)
Temperature (K)	100
a, *b*, *c* (Å)	7.2023(6), 11.2757(10), 12.4290(13)
α, *β*, *γ* (°)	111.659(9), 103.928(8), 91.082(7)
V (Å3)	904.01(16)
Z	2
Density (calcd.) (g/cm^3)	1.511
μ (mm^{-1})	0.221
F(000)	424
Crystal size (mm^3)	0.25 × 0.02 × 0.02
q (º) for data collection	25.242 to 27.997
Completeness	0.999
Absorption correction	multi-scan
T_{min}, T_{max}	0.686,1.000
Reflections/restraints/parameters	4365/0/265
Goodness-of-fit on *F*2	0.997
R(reflections) [*I*>2s(*I*)]	0.0663
*w*R2(reflections)	0.1445

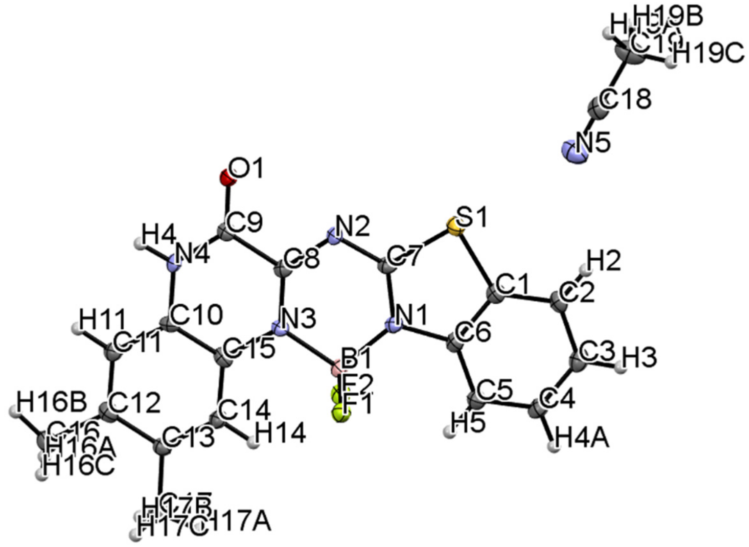

Figure S6. X-ray crystal structure of **4a** with labels.

Table S2. Bond length for **4a**.

Atom	Atom	Length/Å	Atom	Atom	Length/Å
S1	C7	1.732(3)	C10	C15	1.389(4)
S1	C1	1.748(3)	C10	C11	1.395(4)
F2	B1	1.376(4)	C15	C14	1.406(4)
F1	B1	1.385(4)	C9	C8	1.503(4)
O1	C9	1.228(3)	C14	C13	1.375(4)
N1	C7	1.333(3)	C6	C1	1.395(4)
N1	C6	1.400(3)	C6	C5	1.386(4)
N1	B1	1.543(4)	C1	C2	1.396(4)
N4	C10	1.391(3)	C12	C13	1.410(4)
N4	C9	1.343(3)	C12	C11	1.377(4)
N2	C7	1.329(3)	C12	C16	1.507(4)
N2	C8	1.338(3)	C2	C3	1.385(4)
N3	C15	1.409(3)	C5	C4	1.378(4)
N3	C8	1.338(3)	C13	C17	1.508(4)
N3	B1	1.577(4)	C4	C3	1.384(4)
N5	C18	1.136(4)	C18	C19	1.450(5)

Table S3. Bond angels for **4a**.

Atom	Atom	Atom	Angle/°	Atom	Atom	Atom	Angle/°
C7	S1	C1	90.07(13)	C1	C6	N1	112.8(2)
C7	N1	C6	113.0(2)	C5	C6	N1	126.3(3)
C7	N1	B1	122.6(2)	C5	C6	C1	120.9(3)
C6	N1	B1	124.3(2)	C6	C1	S1	110.5(2)
C9	N4	C10	124.5(2)	C6	C1	C2	121.2(3)
C7	N2	C8	117.2(2)	C2	C1	S1	128.3(2)
C15	N3	B1	117.6(2)	C13	C12	C16	120.4(3)
C8	N3	C15	120.8(2)	C11	C12	C13	118.9(3)
C8	N3	B1	121.6(2)	C11	C12	C16	120.8(3)
N4	C10	C11	120.7(3)	C3	C2	C1	116.9(3)
C15	C10	N4	118.9(3)	C4	C5	C6	117.9(3)
C15	C10	C11	120.4(3)	C14	C13	C12	119.6(3)
C10	C15	N3	119.7(2)	C14	C13	C17	119.9(3)
C10	C15	C14	117.9(3)	C12	C13	C17	120.5(3)
C14	C15	N3	122.4(3)	C5	C4	C3	121.3(3)
O1	C9	N4	123.2(3)	N5	C18	C19	179.2(3)
O1	C9	C8	121.5(3)	C12	C11	C10	121.3(3)
N4	C9	C8	115.3(2)	C4	C3	C2	121.8(3)
N1	C7	S1	113.6(2)	F2	B1	F1	110.5(2)
N2	C7	S1	120.1(2)	F2	B1	N1	109.9(2)
N2	C7	N1	126.4(3)	F2	B1	N3	110.9(2)
N2	C8	N3	126.0(3)	F1	B1	N1	109.8(2)
N2	C8	C9	113.4(2)	F1	B1	N3	109.3(2)
N3	C8	C9	120.6(3)	N1	B1	N3	106.3(2)
C13	C14	C15	121.9(3)				

iii. Photophysical properties

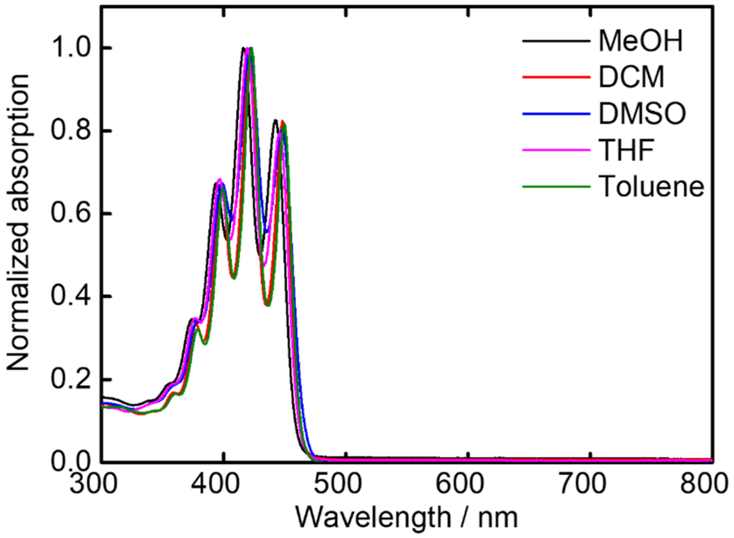

Figure S7. UV/vis absorption spectra of **4a** in various solvents.

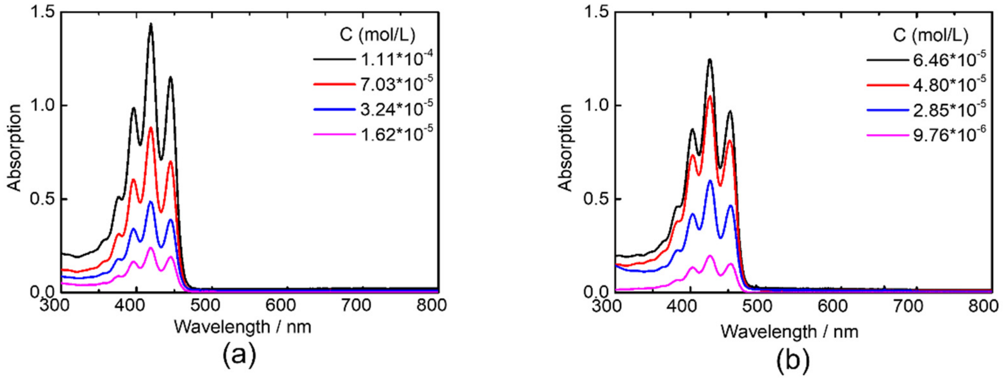

Figure S8. UV/vis absorption spectra of **4a** (a) and **4b** (b) in THF at different concentrations.

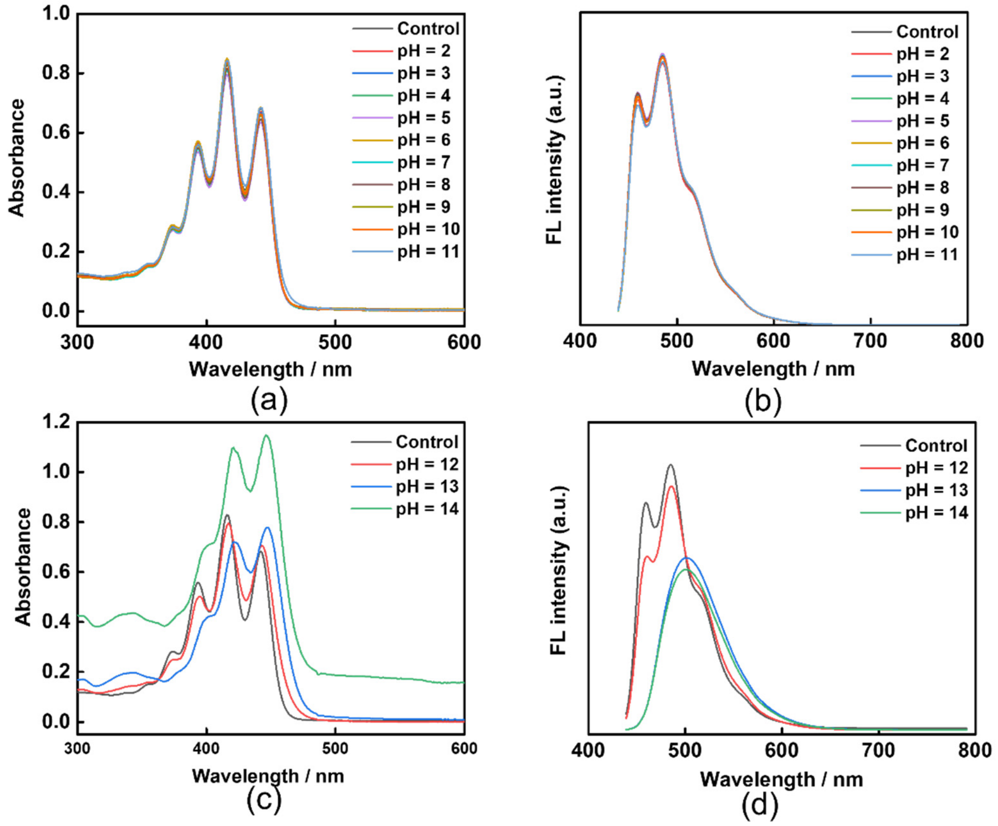

Figure S9. UV/vis absorption and fluorescence spectra of **4a** at various pH values in CH_3CN solution.
